# Complement 5a is an indicator of significant fibrosis and earlier cirrhosis in patients chronically infected with hepatitis B virus

**DOI:** 10.1007/s15010-016-0942-7

**Published:** 2016-09-07

**Authors:** Yongqiong Deng, Hong Zhao, Jiyuan Zhou, Linlin Yan, Guiqiang Wang, China HepB-Related Fibrosis Assessment Research Group

**Affiliations:** 1 Department of Infectious Disease, Center for Liver Disease, Peking University First Hospital, No. 8, Xishiku Street, Xicheng District, Beijing, 100034 China; 2 The Collaborative Innovation Center for Diagnosis and Treatment of Infectious Diseases, Zhejiang University, Hangzhou, Zhejiang China; 3 The Department of Dermatology, The Affiliated Hospital of Southwest Medical University, Luzhou, Sichuan China; 4 The Coordination Innovation Center, Ministry of Education, Beijing, China

**Keywords:** Complement 5a, Hepatitis B, Liver fibrosis, Cirrhosis

## Abstract

**Purpose:**

To investigate the association between serum complement 5a (C5a) concentration and liver fibrosis and cirrhosis in a large cohort of patients chronically infected with hepatitis B virus (HBV).

**Methods:**

Five hundred and eight patients with chronic HBV infection undergoing liver biopsy were included. Serum concentrations of C5a was measured by Luminex screening system. Ishak histological system was obtained.

**Results:**

C5a levels were negatively associated with liver fibrosis stages and significantly declined in patients with severe fibrosis and cirrhosis (*P* < 0.001). Multiple analysis showed C5a, AST, laminin, Co-IV, platelet count, albumin, HBsAg associated with liver fibrosis independently. Based on the markers above, we created two scores, Fib-model for significant fibrosis and Cirrh-model for earlier cirrhosis. Fib-model was performing better to differentiate from significant fibrosis, with an AUROC of 0.82 (95 % CI 0.78, 0.86), in comparison to existed models APRI, FIB-4 and Forns’ index with AUROCs of 0.71 (95 % CI 0.66, 0.76), 0.72 (95 % CI 0.67, 0.77), 0.77 (95 % CI 0.72, 0.81), respectively. Although, Cirrh-model showed AUROC of 0.85 (95 % CI 0.80, 0.91) for evaluation of earlier cirrhosis, superior to APRI, and Forns’ index, C5a + FIB-4 performed best with an AUROC of 0.94 (95 % CI 0.90, 0.97).

**Conclusion:**

In patients with chronic HBV infection, serum C5a concentration significantly decreased in severe fibrosis stages and earlier cirrhosis. Fib-model and C5a + FIB-4 performed better than existed models for assessment of significant fibrosis and earlier cirrhosis, respectively.

**Electronic supplementary material:**

The online version of this article (doi:10.1007/s15010-016-0942-7) contains supplementary material, which is available to authorized users.

## Introduction

Chronic HBV infection was a serious health problem affecting approximately 5 % of the world’s population, which could further develop cirrhosis and hepatocellular carcinoma (HCC) [1]. Liver fibrosis was part of the natural wound healing response to parenchymal injury and generally considered to be a key event resulting in cirrhosis. The accurate assessment of liver fibrosis staging should enable clinical physicians to determine individual management and monitor the disease progression. Liver biopsy remained the gold standard for assessing fibrosis and cirrhosis, however, up to 2 % of patients develop complications from this procedure [2]. The cost, invasiveness and risks associated with liver biopsy limited its use for disease assessment and monitoring.

In the past decade, non-invasive methods for assessment of liver fibrosis have been developed as surrogates to liver biopsy, based on a “biological” approach (quantifying biomarkers in serum samples) or based on a “physical” approach (measuring liver stiffness) [3, 4]. Compared with liver stiffness measurement, serum biomarkers included such advantages as high applicability (95 %), good inter-laboratory reproducibility and widespread availability [5–7].

Complement 5 (C5), a serum protein that is an integral component of the complement activation cascade, generate two distinct products upon proteolytic leavage: C5b leading to the formation of a lytic membrane attack complex (MAC), and C5a [8]. C5a was first described as an anaphylatoxin and later as a leukocyte chemoattractant, and recently was also implicated in non-immunological functions associated with development biology, neurodegeneration, tissue regeneration, and haematopoiesis [9]. In liver, C5a receptor (C5aR) was shown to be universally expressed on the surface of Rat Kupffer (KC), hepatic stellate cell (HSC) and sinusoidal endothelial cells (SEC), which were known to play a key role in the induction of liver fibrosis [10]. The Study by Xu Ruonan and their colleagues indicated that C5a significantly activated HSCs and up-regulated α-smooth muscle actin, hyaluronic acid and type IV collagen expression [11]. Another study showed up-regulation of fibronectin but not entactin, collagen IV and smooth muscle actin by anaphylatoxin C5a in rat HSCs [10]. It was reported that small molecule inhibitors of the C5a receptor had antifibrotic effects in vivo, and common haplotype-tagging polymorphisms of the human gene C5 were associated with advanced fibrosis in chronic hepatitis C virus infection [12]. Recently, plasma C5a concentration has been reported increasing in 73 chronic hepatitis B patients than in 17 healthy control subjects, particularly in those patients with higher inflammation grade and fibrosis stage [11]. However, the small sample size was the main drawback of the study. Additionally, the cohort contained some patients who were receiving antiviral treatment, which may contribute to inaccurate analysis. In fact, the performance of C5a as an potential biomarker for predicting liver fibrosis stages has not been fully identified.

This study was designed to investigate the association between serum C5a concentration and liver fibrosis and cirrhosis in a large cohort of patients chronically infected with HBV.

## Patients and methods

### Patients

This study included 508 patients with chronic HBV infection from 24 hospitals described previously [13] in mainland of China between October of 2014 and October of 2015 and 18 patients from Southwest medical University T.C.M hospital. All patients were recruited for China HepB Related Fibrosis Assessment Research supported by China Mega-project for Infectious Diseases. Inclusion and exclusion criteria were previously described [13]. All patients gave written informed consent to entry to the project for use of clinical data and specimens for research purpose. The full, detailed clinical trials protocol was registered at clinicaltrials.gov (NCT01962155) and chictr.org (ChiCTR-DDT-13003724). The study was approved by The Ethical Committees of Peking University First Hospital.

### Laboratory test

Biochemical and hematological parameters including platelet counts, alanine transaminase (ALT), aspartate aminotransferase (AST), alkaline phosphatase (ALP) gamma-glutamyltransferase (GGT), albumin, total bilirubin (TBil), prothrombin time (PT), cholesterol were routinely detected by standard assays and methods in local hospitals. Clinical, biochemical, and hematological data were recorded from each patient within 4 weeks prior to liver biopsy. Non-invasive fibrosis scores were calculated according to the following formulae: APRI = ([AST/ULN]/platelet count [×10^9^/L]) × 100; FIB-4 = (age × AST)/(platelet count) [×10^9^/L] × ALT^1/2)^; and Forns’ index = 7.811 − 3.131 × LN (platelet count) + 0.781 × LN (GGT) + 3.467 × LN (age) − 0.014 × LN (cholesterol) [14–16]. The blood sample was taken at the time of liver biopsy and stored at −80 °C.

Serum HBsAg levels (rang of 20–52000 IU/ml) were quantified using the Roche Elecsys HBsAg II assay (Roche Diagnostics, Penzberg, Germany) and HBV-DNA (range 2.0 × 10^1^–1.7 × 10^8^ IU/ml) was measured by COBAS AmpliPrep/COBAS TagMan, Roche Diagnostics, Basel, Switzerland.

The serum concentrations of C5a and collagen IV (CO-IV) were measured using the Human Diagnostic Luminex Screening System (LXSAHM-6, R&D Systems, Inc, Minneapolis, MN, USA) according to the manufacturer’s instructions. Concentrations of laminin (5–900 μg/L), hyaluronic acid (range of 2–200 μg/L), procollagen type III N-terminal peptide (PIIINP) (range from 6 to 1000 μg/L) were detected using a chemiluminescent quantitative immunoassay (The source, biomedical engineering co., LTD, Beijing, China). The coefficient of variation (CV) between the duplicate wells was controlled within 10 % and *R*-square of the standard curve was at least 0.999.

### Histology

Liver biopsy and histopathological examination were performed as previously reported [13]. All biopsies had a minimal length of 20 mm (with at least 11 portal tracts) and were scored according to Ishak system [17]. Nil/mild fibrosis was defined as F0-1, moderate fibrosis as F2, significant fibrosis as F3, severe fibrosis as F4 and cirrhosis as *F* ≥ 5.

### Statistics

One-way analysis of variance (ANOVA) was used for comparison of multiple groups. Differences of normally and non-normally distributed variables between the groups were analyzed using Student t test, Kruskal–Wallis and Mann–Whitney *U* tests, respectively. To assess differences in proportions, Chi-square test was used. We performed multiple ordered logistic regression analyses with Ishak fibrosis score as the dependent variable and parameters as the explanatory to compute regression equations. Receiver operating characteristics (ROC) curves were created for the assessment of non-invasive models for staging liver fibrosis and cirrhosis. The predictive performance expressed as areas under the ROC (AUCROCs), sensitivity, specificity, positive predictive value (PPV), and negative predictive value (NPV). The classification accuracy of variables for diagnosis was validated via leave-one-out cross-validation (LOOCV). All data were expressed as the mean ± standard deviation (SD) or proportions, and *P* < 0.05 were considered statistically significant. SPSS 17.0 was used for data analysis.

## Results

### Patients characteristics

Twenty two patients were excluded because of liver biopsy length less than 20 mm and/or 11 portal tracts, and 486 cases with chronic HBV infection were analyzed finally. Basic demographic, clinical and biochemical data of patients were presented in Table 1. The mean age of cohort was 38.91 ± 10.63 years and 377 (77.6 %) were male. One hundred and eighty seven (38.5 %) patients were histologically classified as at least significant fibrosis and 31 (6.4 %) ones as cirrhosis. Parameters of age, ALT, AST, ALP, GGT, Total bilitubin, hyaluronic acid, laminin, PIIINP and CO-IV were positively, and platelet count, albumin, Lg HBVDNA, lg HBsAg were negatively associated with fibrosis stages. In the group of cirrhosis, 29 (90.3 %) patients were diagnosed as earlier fibrosis (*F* = 5). Compared with severe fibrosis, earlier fibrosis did not showed lower platelet count and albumin significantly.

**Table 1:** Patients characteristics

Parameters	F0-1 (*n* = 148, 30.5 %)	F2 (*n* = 151, 31.1 %)	F3 (*n* = 90, 18.5 %)	F4 (*n* = 66, 13.6 %)	F5–6 (*n* = 31, 6.4 %)	*P*
Age (≥40 years, n)	47 (31.8 %)	66 (43.7 %)	50 (55.6 %)	35 (53.0 %)	18 (58.1 %)	
Gender (male, *n*)	114 (77.0 %)	118 (78.1 %)	65 (72.2 %)	57 (86.4 %)	23 (74.2 %)	
BMI (Kg/m^2^)	22.84 ± 2.48	23.49 ± 4.46	23.53 ± 2.86	24.24 ± 2.86	23.67 ± 3.43	0.073
Platelet count (×10^9^/L)	192.95 ± 53.92	184.65 ± 55.04	152.84 ± 49.10	138.42 ± 44.56	131.00 ± 60.14	<0.001
ALT (U/L)	72.27 ± 85.11	123.75 ± 143.19	119.54 ± 181.15	125.79 ± 210.96	97.49 ± 76.76	0.049
AST (U/L)	43.73 ± 41.05	74.41 ± 79.77	89.35 ± 131.05	80.51 ± 120.72	72.64 ± 40.58	0.002
GGT (U/L)	78.32 ± 25.34	80.21 ± 24.77	90.97 ± 35.48	94.16 ± 27.87	100.50 ± 43.17	<0.001
ALP (U/L)	35.34 ± 40.21	54.66 ± 64.43	69.47 ± 63.14	83.30 ± 74.81	78.21 ± 79.99	<0.001
Albumin (g/L)	45.47 ± 5.26	44.56 ± 4.53	43.47 ± 5.79	42.71 ± 6.12	39.12 ± 6.77	<0.001
Total bilirubin (µmol/L)	16.48 ± 21.06	16.30 ± 15.49	17.13 ± 9.69	18.74 ± 9.59	26.54 ± 22.59	<0.002
PT (s)	13.62 ± 11.27	13.35 ± 6.63	13.36 ± 1.53	13.27 ± 2.10	13.67 ± 1.87	0.999
HBV DNA (log_10_IU/ML)	6.85 ± 1.97	6.26 ± 1.80	6.11 ± 1.64	5.77 ± 1.93	5.97 ± 1.81	<0.001
HBsAg (log_10_IU/ML)	3.98 ± 0.96	3.63 ± 0.82	3.19 ± 0.92	3.24 ± 0.62	3.34 ± 0.54	<0.001
HA (μg/L)	107.20 ± 56.70	118.91 ± 57.06	159.62 ± 103.67	170.29 ± 90.46	218.47 ± 183.39	<0.001
LN (μg/L)	44.87 ± 122.13	94.51 ± 197.49	173.34 ± 243.91	288.46 ± 393.37	173.00 ± 213.16	<0.001
PIIINP (μg/L)	3.21 ± 3.74	4.54 ± 7.83	6.31 ± 16.13	6.34 ± 6.17	5.67 ± 4.01	<0.001
CO-IV (log_10_pg/ml)	2.86 ± 0.16	2.90 ± 0.23	3.02 ± 0.22	3.06 ± 0.25	3.08 ± 0.21	<0.001

*F* fibrosis stage, *BMI* body mass index, *ALT* alanine transaminase, *AST* aspartate transaminase, *ALP* alkaline phosphatase, *GGT* gamma-glutamyltransferase, *PT* prothrombin time, *HBV* hepatitis B virus, *HBsAg* HBV surface antigen, *HA* hyaluronic acid, *LN* laminin, *PIIINP* procollagen III N-terminal peptide, *CO-IV* collagen IV

### C5a as predictor for fibrosis and earlier cirrhosis

**Fig. 1:**
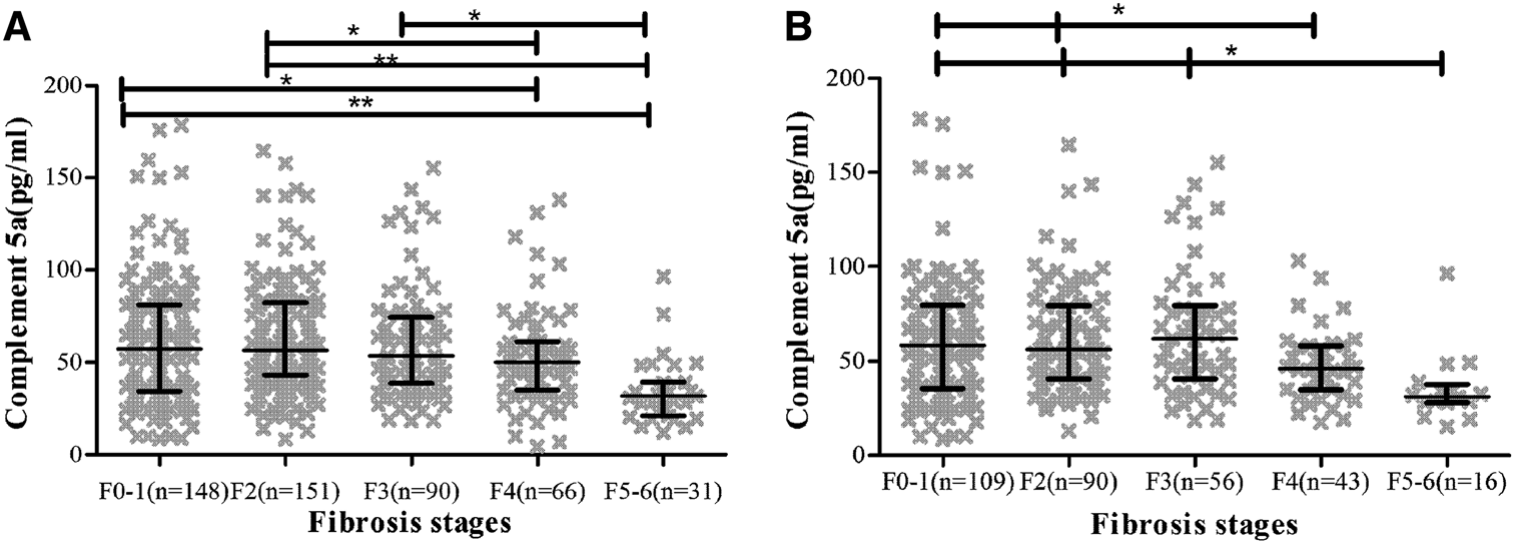
Association between complement 5a concentration and liver fibrosis. *Dotplots* for complement 5a according to fibrosis stage showing mean values and interquartile ranges (IQRs). **a** Complement 5a in total patients; **b** complement 5a in patients with ALT ≤ 2 × ULN. *P* < 0.001 for all fibrosis stags. ***p < 0.001, **p < 0.01, *p < 0.05

In patients with nil/mild fibrosis, C5a was median 67.83(SD 64.17); in moderate fibrosis stage, 73.97 (SD 74.56); in significant fibrosis stage, 62.23 (SD 41.45); in severe fibrosis stage, 51.85 (SD 27.30); in cirrhotic stage, 34.66 (SD 17.89); *P* < 0.001. C5a levels significantly decreased in patients with severe fibrosis and cirrhosis. Figure 1 showed C5a levels throughout different fibrosis stages. In patients with ALT less than 2 times of upper limit (ULN), C5a levels were also negatively associated with liver fibrosis stages and significantly declined in patients with severe fibrosis and cirrhosis.

### Development of C5a -based scores for assessing significant fibrosis and earlier cirrhosis

We then performed multiple ordered logistic regression analyses with Ishak fibrosis score as the dependent variable and all possible parameters above as the explanatory, and used the coefficients (*β*) from the regression equations to compute and examine all possible predictive models. The presence of significant fibrosis (*F* ≥ 3) was usually used as a determinant for initiating antiviral therapy, and cirrhosis (*F* ≥ 5) indicated the need for screening HCC. AS the predictive models including C5a, AST, Laminin, Co-IV, Platelet count, Albumin, HBsAg had the highest AUROCs for significant fibrosis and cirrhosis, we choose the two models as the novel C5a-based fibrosis scores in patients chronically infected with HBV. The coefficients and the odds with 95 % confidence interval of such selected parameters from the two regression equation for predicting significant fibrosis and cirrhosis were shown in Table 2.

**Table 2:** Multiple ordered logistic regression analysis with Ishak fibrosis stages as the dependent variable in patients with chronic hepatitis B

Variable	Fib-model			Cirrh-model		
	*β*	OR (95 % CI)	*P*	*β*	OR (95 % CI)	*P*
C5a	−0.013	0.987 (0.976, 0.999)	0.029	−0.045	0.956 (0.932, 0.980)	<0.001
logCO-IV	1.832	6.249 (0.749, 52.159)	0.091	3.686	39.900 (2.320, 686.252)	0.011
logHBsAg	−0.948	0.388 (0.243, 0.617)	<0.001	−0.582	0.559 (0.286, 1.091)	0.088
Albumin	−0.046	0.955 (0.884, 1.031)	0.234	−0.198	0.820 (0.734, 0.917)	<0.001
Platelet count	−0.017	0.984 (0.976, 0.991)	<0.001	−0.016	0.984 (0.975, 0.994)	0.002
AST	0.006	1.006 (0.999, 1.013)	0.090	0.003	1.003 (0.994, 1.013)	0.463
LN	0.004	1.004 (1.001, 1.007)	0.012	0.001	1.001 (0.997, 1.004)	0.699

*OR* odds ratio, *CI* confidence interval, *C5a* complement 5a, *CO-IV* collagen IV, *HBsAg* HBV surface antigen, *AST* aspartate transaminase, *LN* laminin

$${\text{G}{\times}{1}} = 2.0 6 5- 0.0 1 3\times {\text{C5a}} + 1. 8 3 2\times { \lg }\left( {\text{Co-IV}} \right) - 0. 9 4 8\times { \lg }\left( {\text{HBsAg}} \right) - 0.0 4 6\times {\text{Albumin}} - 0.0 1 7\times {\text{PLT}} + 0.00 6\times {\text{AST}} + 0.00 4\times {\text{Laminin}}$$

$${\text{Fib-model}} = { \exp }\left( {\text{gx1}} \right)/\left[ { 1+ { \exp }\left( {\text{gx1}} \right)} \right]$$

$${\text{G}{\times}{2}} = 2. 6 90 - 0.0 4 5\times {\text{C5a}} + 3. 6 8 6\times { \lg }\left( {\text{Co-IV}} \right) - 0. 5 8 2\times { \lg }\left( {\text{HBsAg}} \right) - 0. 1 9 8\times {\text{Albumin}} - 0.0 1 6\times {\text{PLT}} + 0.00 3\times {\text{AST}} + 0.00 1\times {\text{Laminin}}$$

$${\text{Cirrh-model}} = { \exp }\left( {\text{gx2}} \right)/\left[ { 1+ { \exp }\left( {\text{gx2}} \right)} \right]$$

### Diagnostic performance of C5a based scores, in comparison to APRI, FIB-4 and Forns’ index

**Fig. 2:**
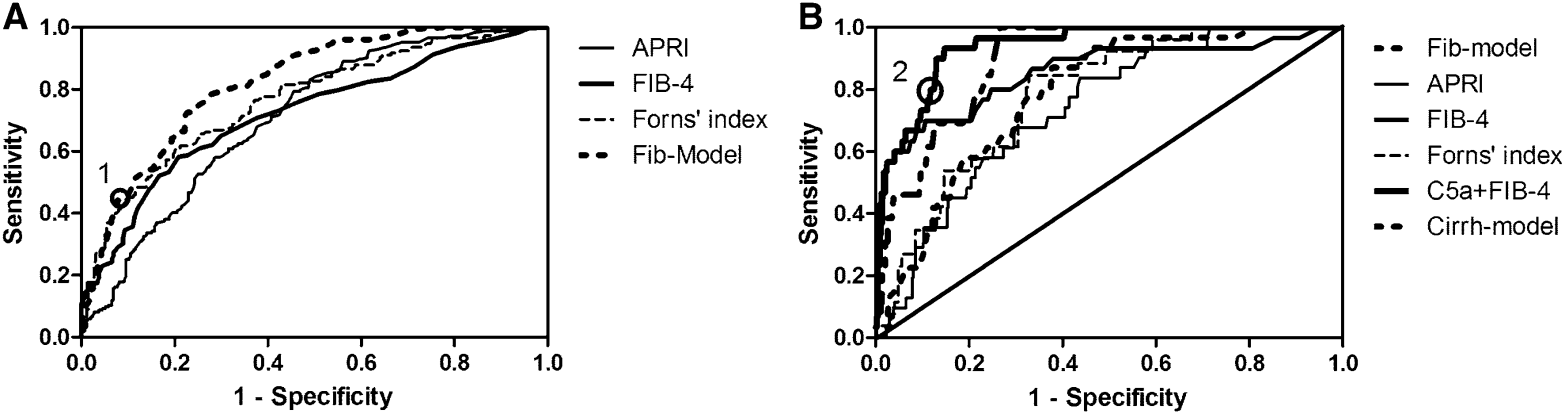
Receiver operating characteristics (ROC) analysis showing the predictive value of non-invasive models for significant fibrosis and cirrhosis. **a** Area under the ROC curves (AUC) for Fibmodel, ARPI, FIB-4, and Forns’ index in the diagnosis of significant fibrosis (*F* ≥ 3): AUC Fibmodel = 0.82 (0.78, 0.86), APRI = 0.71 (0.66, 0.76), FIB-4 = 0.72 (0.67, 0.77), Forns’ index = 0.77 (0.72, 0.81). (Marker 1: cut off at 0.67). **b** Area under the ROC curves (AUC) for Fib-model, ARPI, FIB-4, Forns’ index and C5a + FIB-4 in the diagnosis of cirrhosis (*F* ≥ 5): AUC Fib-model = 0.79 (0.72, 0.85), APRI = 0.74 (0.67, 0.82), FIB-4 = 0.85 (0.77, 0.94), Forns’ index = 0.78 (0.71, 0.86), C5a + FIB-4 = 0.94 (0.90, 0.97).(Marker 2: cut off at −2.625)

Table 3 showed the diagnostic performance of non-invasive models predicting liver fibrosis. Fib-model was performing best in our group to differentiate from significant fibrosis, with an AUROC of 0.82 (95 % CI 0.78, 0.86), in comparison to existed models APRI, FIB-4 and Forns’ index with AUROCs of 0.71 (95 % CI 0.66, 0.76), 0.72 (95 % CI 0.67, 0.77), 0.77 (95 % CI 0.72, 0.81), respectively. When C5a was combined with APRI, FIB-4 and Forns’ index for assessment of significant fibrosis, AUROCs were not enhanced significantly. We identified cutoff value for Fib-model for the presence or absence of significant fibrosis, based on the ROC-curve (Fig. 2a). The cutoff for significant fibrosis at Fib-model was 0.67 (Marked 1 on Fig. 2a), with a sensitivity of 44.1 %, specificity of 92.3 %, PPV of 82.0 %, NPV of 76.8 %.

For evaluation of cirrhosis, C5a + FIB-4 performed best with an AUROC of 0.94 (95 % CI 0.90, 0.97). FIB-4 and Cirrh-model with AUROCs of 0.85 (95 % CI 0.77, 0.94) and 0.85 (95 % CI 0.80, 0.91) were performing as the second best (Fig. 2b). The cutoff value of C5a + FIB-4 for cirrhosis was −2.625 (marked 2 on Fig. 2b), with a sensitivity of 80 %, a specificity of 88.2 %, a PPV of 85.8 % and a NPV of 82.9 %. With C5a + FIB-4, we correctly diagnosed 83.8 % of the patients with cirrhosis.

**Table 3:** Areas under receiver operating characteristics (AUROCs) of non-invasive models for liver fibrosis

Models	AUROC (95 % CI)		
	F0-2 VS F3-6	F0-3 VS F4-6	F0-4 VS F5-6
APRI	0.71 (0.66, 0.76)	0.70 (0.65, 0.77)	0.74 (0.67, 0.82)
FIB4	0.72 (0.67, 0.77)	0.72 (0.65, 0.78)	0.85 (0.77, 0.94)
Forns’ index	0.77 (0.72, 0.81)	0.77 (0.71, 0.82)	0.78 (0.71, 0.86)
Fib-model	0.82 (0.78, 0.86)	0.82 (0.78, 0.86)	0.79 (0.72, 0.85)
Cirrh-model	0.78 (0.74, 0.83)	0.80 (0.75, 0.85)	0.85 (0.80, 0.91)
C5a + APRI	0.72 (0.65, 0.79)	0.74 (0.69, 0.79)	0.81 (0.75, 0.88)
C5a + FIB4	0.74 (0.69, 0.78)	0.73 (0.67, 0.79)	0.94 (0.90, 0.97)
C5a + Forns’ index	0.78 (0.734, 0.826)	0.79 (0.75, 0.84)	0.82 (0.76, 0.88)

*CI* confidence interval, *C5a* complement 5a

To validate these non-invasive models for predicting significant fibrosis and cirrhosis, LOOCV was performed. For significant fibrosis, LOOCV showed that 73.3 % cross-validation grouped cases were correctly classified by Fib-model and 64.8, 70.4, and 67.1 % classified by APRI, FIB-4 and Forns’ index (Supplemental Table S1). LOOCV also showed that C5a + FIB-4 could correctly classified 89.3 % of cases, and Cirrh-model, APRI,FIB-4 and Forns’ index just showed 71.4, 78.4, 82.6 and 75.1 % correct classification (Supplemental Table S1).

## Discussion

Increasing evidence indicated that C5a participated in the pathogenesis of liver disorders, including liver injury, repair, and fibro-genesis. Complement 5, shown earlier to be correlated with liver fibrosis in mice, was found to be elevated in MTX-exposed livers [18]. C5a also could act as a growth factor in regenerating rat hepatoyes under inflammatory conditions [19]. C5-deficient mice showed impairment of liver regeneration and persistent parenchymal necrosis after exposure to CCL4 and reconstitution of C5-deficient mice with C5a significantly restored hepatocyte regeneration in a course of 6–7 days [20]. The above observations highlight C5a as one among essential factors that mediate liver regeneration and that it probably exerted its function in an early stage during this process. In this study including 486 patients, serum concentration of C5a declined significantly in severe fibrosis and earlier cirrhosis stage, which was similar to the change of another complement C4a in liver fibrogenesis. C4a was primarily expressed in liver and could induce in response to acute inflammations or tissue injury [21]. In patients with chronic hepatitis C, C4a and C3 were found to decrease in advanced fibrosis and cirrhosis [22–25]. Additionally, 90 % of complement was synthesized by liver, the significant decreasing of C5a may result from the dysfunction of hepatocyte in severely fibrotic and cirrhotic liver.

Detection of significant fibrosis (Ishak, *F* ≥ 3) and cirrhosis (Ishak, *F* ≥ 5) were the most important clinically relevant endpoints in patients with chronic hepatitis B. A diagnosis of significant fibrosis indicated that patients should receive antiviral treatment [26, 27]. However, throughout the articles, serum biomarkers for non-invasive assessment of liver fibrosis were better for detecting cirrhosis than significant fibrosis. Serum biomarkers and TE showed to have equivalent performance for detecting significant fibrosis [28–30]. The most widely validated serum biomarkers in chronic hepatitis B patients were APRI, FIB-4. A meta-analysis for APRI in 1798 HBV patients found mean AUROC values of 0.79 for significant fibrosis. In this study,the performance of C5a based model fib-model was superior to APRI, FIB-4 and Forns’ index for predicting significant fibrosis.

Once diagnosis of cirrhosis has been established, AASLD and EASL guidelines recommended that patients should be monitored for complications related to portal hypertension and regularly screened for HCC [26, 27]. In fact, it is more important to detect earlier cirrhosis than decompensated cirrhosis, which was not easily be found only according to hematologic, biochemical tests or abdominal ultrasound. In this study, decompensated cirrhosis was one of the exclusions, earlier fibrosis (*F* = 5) diagnosed by histology in 28 (90.3 %) patients in the group of cirrhosis. The concentration of C5a declined in earlier cirrhosis and helped the diagnosis. AUROC of combination of C5a and FIB-4 for predicting earlier cirrhosis was 0.94, significantly superior to APRI, FIB-4 and Forn’s index.

Although transient elastography (TE) more accurately detected cirrhosis (AUROC values 0.88–0.99), it is usually only available in specialized centres and its applicability is not as good as that of serum biomarkers [3, 31]. Besides, TE values have been reported overestimation due to ALT flares [3]. Another limitation for using TE seems the difficulty to obtain from obese patients, and patients with ascites [32].

One clear limitation of this study was the small sample size of cirrhotic patients, the value of C5a for diagnosing earlier cirrhosis should be validated in a large cohort in future study. The second limitation was that a substantial overlap of C5a concentration was observed between adjacent stages of the fibrosis, especially for lower fibrosis stages. In fact, other excellent biomarkers reported in previous study also failed to avoid overlap [31, 33]. Although lack of validation group was another limitation of this study, LOOCV which has the advantages of producing model estimates more easily and with less bias in smaller samples has been used to cover the shortage [34]. Additionally,this study is clinical research. The basic research of the relationship between Angptl2 and liver fibrosis is currently in progress.

In conclusion, C5a declined significantly in severe fibrotic and early cirrhotic stages in patients with chronic HBV infection. Combination of C5a and FIB-4 offered an easy possibility to diagnose early cirrhosis and its performance was superior to existing scores APRI, FIB-4 and Forns’ index.

## Electronic supplementary material

Below is the link to the electronic supplementary material.

Supplementary material 1 (DOCX 13 kb)
