# Supplementary material for: Complement 5a is an indicator of significant fibrosis and earlier cirrhosis in patients chronically infected with hepatitis B virus
Source: Infection. 2016 Sep 7;45(1):75–81. doi: 10.1007/s15010-016-0942-7 (PMC5306372; doi:10.1007/s15010-016-0942-7)
Supplement: Supplementary file 1 — Supplementary material 1 (DOCX 13 kb) [file 15010_2016_942_MOESM1_ESM.docx]

Supplement Table 1. Areas under the Receiver Operating Characteristics Curve and Classification Accuracy of Inflammation-index for moderate inflammation, and of fib-index,FIB-4 score,APRI for significant fibrosis.

| Models | Total Patients | | | Patients with ALT＜2×ULN | | |
| --- | --- | --- | --- | --- | --- | --- |
|  | AUC | 95% CI | Classification accuracy(%) | AUC | 95% CI | Classification accuracy(%) |
| For significant fibrosis |  |  |  |  |  |  |
| Fib-model | 0.82 | 0.78, 0.86 | 73.3 | 0.82 | 0.78, 0.87 | 75.2 |
| APRI | 0.71 | 0.66, 0.76 | 64.8 | 0.79 | 0.74, 0.84 | 72.0 |
| FIB-4 | 0.72 | 0.67, 0.77 | 70.4 | 0.75 | 0.69, 0.81 | 70.1 |
| Forns’ index | 0.77 | 0.72, 0.81 | 67.1 | 0.78 | 0.73, 0.84 | 68.5 |
| For cirrhosis |  |  |  |  |  |  |
| Cirrh-model | 0.79 | 0.72, 0.85 | 71.4 | 0.76 | 0.67, 0.85 | 71.7 |
| APRI | 0.74 | 0.67, 0.82 | 78.4 | 0.78 | 0.68, 0.87 | 82.5 |
| FIB-4 | 0.85 | 0.77, 0.94 | 82.6 | 0.85 | 0.75, 0.95 | 81.1 |
| Forns’ index | 0.78 | 0.71, 0.86 | 75.1 | 0.81 | 0.71, 0.91 | 76.8 |
| C5a+ FIB-4 | 0.94 | 0.90, 0.97 | 89.3 | 0.92 | 0.87, 0.96 | 86.0 |
